# Supplementary material for: Developing and validating a HEalthCare NAvigation Competency (HECNAC) Scale for refugees in the United States
Source: PLoS One. 2025 Jan 30;20(1):e0314057. doi: 10.1371/journal.pone.0314057 (PMC11781618; doi:10.1371/journal.pone.0314057)
Supplement: S1 Appendix — (DOCX) [file pone.0314057.s001.docx]

# **S1 Appendix. THE FIRST DRAFT OF THE HEALTHCARE NAVIGATION COMPETENCY SCALE**

**Health system knowledge**

| ***When you have the following conditions or symptoms, what should you do?*** | Treat at home | | Go to a primary care (family) doctor) | Go to an urgent care | | Go to emergency room | | Call 911 | | Unsure/  don’t know |
| --- | --- | --- | --- | --- | --- | --- | --- | --- | --- | --- |
| 1. I have difficulty breathing along with chest pain. |  | |  |  | |  | |  | |  |
| 1. I or my children have a mild fever and runny nose. |  | |  |  | |  | |  | |  |
| 1. I think my arm is broken without bleeding or deformation. |  | |  |  | |  | |  | |  |
| 1. My child needs to be vaccinated. |  | |  |  | |  | |  | |  |
| 1. For the last 6 months, I have experienced stomach pain and constipation. |  | |  |  | |  | |  | |  |
| 1. I think I (or my wife) got pregnant. |  | |  |  | |  | |  | |  |
|  | Strongly disagree | Disagree | | | Neither agree  or disagree | | Agree | | Strongly  agree | |
| 1. To see a specialist doctor, I first need to see my primary care doctor (family doctor). |  |  | | |  | |  | |  | |
| 1. I have a family doctor. |  |  | | |  | |  | |  | |

**Insurance**

|  | Strongly disagree | Disagree | Neither agree  or disagree | Agree | Strongly  agree |
| --- | --- | --- | --- | --- | --- |
| 1. I can go to the hospital for all health needs at no cost. |  |  |  |  |  |
| 1. I know where to look when I am unsure about which services are and aren't covered by my insurance. |  |  |  |  |  |
| 1. I can get most preventive care (such as immunization, cancer screening) for free with my insurance. |  |  |  |  |  |

**Making an appointment**

|  | Strongly disagree | Disagree | Neither agree  or disagree | Agree | Strongly  agree |
| --- | --- | --- | --- | --- | --- |
| 1. I know where to call to make a medical appointment with a family doctor. |  |  |  |  |  |
| 1. I know how to call and make a medical appointment. |  |  |  |  |  |
| 1. I have someone who can help me make a medical appointment when needed. |  |  |  |  |  |

**Transportation**

|  | Strongly disagree | Disagree | Neither agree  or disagree | Agree | Strongly  agree |
| --- | --- | --- | --- | --- | --- |
| 1. I can go to a clinic either using public transportation or using my car. |  |  |  |  |  |
| 1. I have someone who can give me a ride to a clinic when needed. |  |  |  |  |  |
| 1. I know what medical taxi is. |  |  |  |  |  |
| 1. I know how to call and schedule a medical taxi when needed. |  |  |  |  |  |

**Preparing for a visit**

|  | Strongly disagree | Disagree | Neither agree  or disagree | Agree | Strongly  agree |
| --- | --- | --- | --- | --- | --- |
| 1. I know the essential documents to take to a medical appointment. |  |  |  |  |  |
| 1. I usually prepare questions to ask for a doctor’s visit. |  |  |  |  |  |
| 1. When I make an appointment, I ask if there are any dietary recommendations before my appointment such as fasting. |  |  |  |  |  |
| 1. When I make an appointment, I ask if there will be a copayment (money that must be paid by the patient) and how much it will be. |  |  |  |  |  |
| 1. (If I have kids) I have someone to watch my kids during my medical appointment if needed. |  |  |  |  |  |

**In the clinic**

|  | Strongly disagree | Disagree | Neither agree  or disagree | Agree | Strongly  agree |
| --- | --- | --- | --- | --- | --- |
| 1. I know how to check in at the reception desk by telling my name or show my ID. |  |  |  |  |  |
| 1. I know how to fill out necessary paperwork with some help. |  |  |  |  |  |
| 1. I can express my concerns to my doctor. |  |  |  |  |  |
| 1. I can ask any questions to my doctor. |  |  |  |  |  |
| 1. I know the location of the pharmacy that is close to my house. |  |  |  |  |  |
| 1. I can let the health care providers know the address of the pharmacy. |  |  |  |  |  |
| 1. At the end of my visit, I ask at the reception desk what to do next (whether I need to return for another visit, need to pick up any medicine, need to go to lab, or get specialist care). |  |  |  |  |  |
| 1. If needed, I know how to get a referral and get specialist care. |  |  |  |  |  |

**Knowing the rights to interpretation services and how to request an interpreter**

|  | Strongly disagree | Disagree | Neither agree  or disagree | Agree | Strongly  agree |
| --- | --- | --- | --- | --- | --- |
| 1. The services of a professional medical interpreter should be provided at no cost to the patients and their family members. |  |  |  |  |  |
| 1. I know how to request an interpreter at clinic, pharmacy, or over the phone. |  |  |  |  |  |

**Medicine**

|  | Strongly disagree | Disagree | Neither agree  or disagree | Agree | Strongly  agree |
| --- | --- | --- | --- | --- | --- |
| 1. I know the process of getting refills for medicine if necessary. |  |  |  |  |  |
| 1. I know how to pick up any prescribed or refilled medicines at the pharmacy. |  |  |  |  |  |
| 1. I know what the over-the-counter medicine is. |  |  |  |  |  |
| 1. I know how to get over-the-counter medicine. |  |  |  |  |  |
| 1. If errors occur at pharmacy - for example, the prescription is not there at the pharmacy, I know what to do and follow up on the issue. |  |  |  |  |  |
| 1. If errors occur at pharmacy - for example, the prescription is not there at the pharmacy, I have someone who can help me solve the issue. |  |  |  |  |  |

**Medical bills**

|  | Strongly disagree | Disagree | Neither agree  or disagree | Agree | Strongly  agree |
| --- | --- | --- | --- | --- | --- |
| 1. I know how to read the medical bills (either myself or using a translating app such as Google translator). |  |  |  |  |  |
| 1. I have someone who can help read the medical bills. |  |  |  |  |  |
| 1. I know how to pay the medical bills if I have money. |  |  |  |  |  |
| 1. If there are any medical billing errors or insurance denies to pay my bills, I know how to address the issues. |  |  |  |  |  |
| 1. If there are any medical billing errors or insurance denies to pay my bills, I have someone who can help me address the issues. |  |  |  |  |  |

**Preventive care**

|  | Strongly disagree | Disagree | Neither agree  or disagree | Agree | Strongly  agree |
| --- | --- | --- | --- | --- | --- |
| 1. Many illnesses can be prevented through cleanliness, proper nutrition, exercise, and adequate sleep. |  |  |  |  |  |
| 1. People at certain ages need to get cancer screening even though they don’t feel sick or don’t have any symptoms. |  |  |  |  |  |
| 1. Vaccinations are effective in preventing some diseases. |  |  |  |  |  |
