# Supplementary material for: Developing and validating a HEalthCare NAvigation Competency (HECNAC) Scale for refugees in the United States
Source: PLoS One. 2025 Jan 30;20(1):e0314057. doi: 10.1371/journal.pone.0314057 (PMC11781618; doi:10.1371/journal.pone.0314057)
Supplement: S2 Appendix — (DOCX) [file pone.0314057.s002.docx]

# **S2 Appendix. THE SECOND VERSION OF THE HEALTHCARE NAVIGATION COMPETENCY SCALE AFTER THE DELPHI**

**Health system knowledge**

| ***When you have the following conditions or symptoms, what should you do?*** | **Treat at home** | **Go to a primary care** | | **Go to an urgent care** | | **Go to an emergency room** | **Call 911** | | **Other** | | **Unsure/**  **don’t know** |
| --- | --- | --- | --- | --- | --- | --- | --- | --- | --- | --- | --- |
| 1. I have difficulty breathing along with chest pain. |  |  | |  | |  |  | |  | |  |
| 1. I have a mild fever (below 100F) and a runny nose. |  |  | |  | |  |  | |  | |  |
| 1. I have experienced occasional stomach pain and constipation for the last 6 months. |  |  | |  | |  |  | |  | |  |
| 1. I need to be vaccinated.   (Treat at home -> I will not get vaccinated  go to primary care or a local pharmacy) |  |  | |  | |  |  | |  | |  |
| 1. I think I am (or my wife is) pregnant. (treat at home = do nothing) |  |  | |  | |  |  | |  | |  |
|  | Strongly disagree | | Disagree | | Neither agree  or disagree | | | Agree | | Strongly  agree | |
| 1. I need to have a primary care provider (family doctor). |  | |  | |  | | |  | |  | |

**Insurance**

|  | Strongly disagree | Disagree | Neither agree  or disagree | Agree | Strongly  agree |
| --- | --- | --- | --- | --- | --- |
| 1. I can go to a clinic for all health needs at no cost. |  |  |  |  |  |
| 1. I know where to learn more or who to ask when I am unsure whether my insurance covers medical treatment. |  |  |  |  |  |
| 1. I can get most preventive care (such as immunization and cancer screening) for free with my insurance. |  |  |  |  |  |

**Making an appointment**

|  | Strongly disagree | Disagree | Neither agree  or disagree | Agree | Strongly  agree |
| --- | --- | --- | --- | --- | --- |
| 1. I know where to call to make a medical appointment with a primary care provider (a family doctor). |  |  |  |  |  |
| 1. I am able to call and make a medical appointment by myself. |  |  |  |  |  |
| 1. I have someone who can help me make a medical appointment when needed. |  |  |  |  |  |

**Transportation**

|  | Strongly disagree | Disagree | Neither agree  or disagree | Agree | Strongly  agree |
| --- | --- | --- | --- | --- | --- |
| 1. I have access to transport to get to my medical appointment. |  |  |  |  |  |

**Preparing for a visit**

|  | Strongly disagree | Disagree | Neither agree  or disagree | Agree | Strongly  agree |
| --- | --- | --- | --- | --- | --- |
| 1. I know the essential documents to take to a medical appointment. |  |  |  |  |  |
| 1. When I make an appointment, I ask if there are any dietary recommendations before my appointment, such as fasting. |  |  |  |  |  |
| 1. When I make an appointment, I ask if there will be a copayment (money that must be paid by the patient) and how much it will be. |  |  |  |  |  |
| 1. (If I have kids) I have someone to watch my kids during my medical appointments if needed.  Not applicable |  |  |  |  |  |

**In the clinic**

|  | Strongly disagree | Disagree | Neither agree  or disagree | Agree | Strongly  agree |
| --- | --- | --- | --- | --- | --- |
| 1. I am able to check in at the reception desk by telling my name and date of birth or showing my ID. |  |  |  |  |  |
| 1. I am able to fill out necessary paperwork by myself, or I have someone who can help with the process. |  |  |  |  |  |
| 1. I feel comfortable discussing my concerns with my health care provider. |  |  |  |  |  |
| 1. I feel comfortable asking any questions to my health care provider. |  |  |  |  |  |
| 1. I know where to go when a prescription is ordered. |  |  |  |  |  |
| 1. I am able to let health care providers know my preferred pharmacy either by telling them or showing my ID. |  |  |  |  |  |
| 1. I know what to expect after my visit and when I should return if necessary. |  |  |  |  |  |
| 1. If needed, I know how to get specialist care. |  |  |  |  |  |

**Interpretation**

|  | Strongly disagree | Disagree | Neither agree  or disagree | Agree | Strongly  agree |
| --- | --- | --- | --- | --- | --- |
| 1. I am able to request an interpreter at a clinic, pharmacy, or over the phone. |  |  |  |  |  |

**Medicine**

|  | Strongly disagree | Disagree | Neither agree  or disagree | Agree | Strongly  agree |
| --- | --- | --- | --- | --- | --- |
| 1. I am able to get refills when I finish medicine if necessary. |  |  |  |  |  |
| 1. I am able to pick up prescribed or refilled medicines at a pharmacy. |  |  |  |  |  |
| 1. I am aware of medications that do not require a prescription from a health care provider (over-the-counter medicine). |  |  |  |  |  |
| 1. I am able to get medications from a pharmacy that do not require a prescription (over-the-counter medicine). |  |  |  |  |  |
| 1. I know what to do when I cannot get my medicine on time (for example, prescription is not at a pharmacy, or something is wrong with the prescription or medication). |  |  |  |  |  |
| 1. I have someone who can help me when I cannot get my medicine on time (for example, prescription is not at a pharmacy, or something is wrong with the prescription or medication). |  |  |  |  |  |

**Medical bills**

|  | Strongly disagree | Disagree | Neither agree  or disagree | Agree | Strongly  agree |
| --- | --- | --- | --- | --- | --- |
| 1. I am able to understand medical bills (either myself or using a translating app). |  |  |  |  |  |
| 1. I have someone who can help me understand medical bills. |  |  |  |  |  |
| 1. I know how to pay medical bills when I need to. |  |  |  |  |  |
| 1. If there are any medical billing errors or insurance declines to pay my bills, I am able to address the issues. |  |  |  |  |  |
| 1. If there are any medical billing errors or insurance declines to pay my bills, I have someone who can help me address the issues. |  |  |  |  |  |

**Preventive care**

|  | Strongly disagree | Disagree | Neither agree  or disagree | Agree | Strongly  agree |
| --- | --- | --- | --- | --- | --- |
| 1. People at certain ages need to get certain tests to check their bodies for possible illnesses like cancer, even if they don’t feel sick. |  |  |  |  |  |
| 1. Vaccinations are effective in preventing some diseases. |  |  |  |  |  |

# 
