# Supplementary material for: Developing and validating a HEalthCare NAvigation Competency (HECNAC) Scale for refugees in the United States
Source: PLoS One. 2025 Jan 30;20(1):e0314057. doi: 10.1371/journal.pone.0314057 (PMC11781618; doi:10.1371/journal.pone.0314057)
Supplement: S3 Appendix — (DOCX) [file pone.0314057.s003.docx]

**S3 Appendix. THE FINAL VERSION OF THE HEALTHCARE NAVIGATION COMPETENCY SCALE**

**Health system knowledge**

Below are some questions to assess your knowledge about the U.S. healthcare system. Read the statements and tell us what actions the person needs to take IF the person experiences these conditions or symptoms. **This person is an adult female**.

|  | **Treat at home** | **Go to a primary care** | | **Go to an urgent care** | | **Go to an emergency room** | **Call 911** | | **Other** | | **Unsure/**  **don’t know** |
| --- | --- | --- | --- | --- | --- | --- | --- | --- | --- | --- | --- |
| 1. She has difficulty breathing along with chest pain. |  |  | |  | |  |  | |  | |  |
| 1. She has a mild fever (below 100F = 37.8C) and a runny nose. |  |  | |  | |  |  | |  | |  |
| 1. She has experienced occasional stomach pain and constipation for the last 6 months. |  |  | |  | |  |  | |  | |  |
| 1. She needs to be vaccinated.   (Treat at home -> one doesn’t need to get vaccinated  go to primary care or a local pharmacy) |  |  | |  | |  |  | |  | |  |
| 1. She is pregnant. (Treat at home = do nothing) |  |  | |  | |  |  | |  | |  |
| **Please read the following statement and indicate your level of agreement with the statement.** | | | | | | | | | | | |
|  | Strongly disagree | | Disagree | | Neither agree  or disagree | | | Agree | | Strongly  agree | |
| 1. I need to have a primary care provider (family doctor). |  | |  | |  | | |  | |  | |

**Insurance**

|  | Strongly disagree | Disagree | Neither agree  or disagree | Agree | Strongly  agree |
| --- | --- | --- | --- | --- | --- |
| 1. I can go to a clinic for all health needs at no cost. |  |  |  |  |  |
| 1. I know where to learn more or who to ask when I am unsure whether my insurance covers medical treatment. |  |  |  |  |  |
| 1. I can get most preventive care (such as immunization and cancer screening) for free with my insurance. |  |  |  |  |  |

**Making an appointment**

|  | Strongly disagree | Disagree | Neither agree  or disagree | Agree | Strongly  agree |
| --- | --- | --- | --- | --- | --- |
| 1. I know where to call to make a medical appointment with a primary care provider (a family doctor). |  |  |  |  |  |
| 1. I am able to call and make a medical appointment by myself or I have someone who can help me make a medical appointment when needed. |  |  |  |  |  |

**Transportation**

|  | Strongly disagree | Disagree | Neither agree  or disagree | Agree | Strongly  agree |
| --- | --- | --- | --- | --- | --- |
| 1. I have access to transport to get to my medical appointment. |  |  |  |  |  |

**Preparing for a visit**

|  | Strongly disagree | Disagree | Neither agree  or disagree | Agree | Strongly  agree |
| --- | --- | --- | --- | --- | --- |
| 1. I know the essential documents to take to a medical appointment. |  |  |  |  |  |
| 1. When I make an appointment, I ask if there are any dietary recommendations before my appointment, such as fasting. |  |  |  |  |  |
| 1. When I make an appointment, I ask if there will be a copayment (money that must be paid by the patient) and how much it will be. |  |  |  |  |  |
| 1. (If I have kids) I have someone to watch my kids during my medical appointments if needed.  Not applicable |  |  |  |  |  |

**In the clinic**

|  | Strongly disagree | Disagree | Neither agree  or disagree | Agree | Strongly  agree |
| --- | --- | --- | --- | --- | --- |
| 1. I am able to check in at the reception desk by telling my name and date of birth or showing my ID. |  |  |  |  |  |
| 1. I am able to fill out necessary paperwork by myself or I have someone who can help with the process. |  |  |  |  |  |
| 1. I feel comfortable discussing my concerns with my healthcare provider. |  |  |  |  |  |
| 1. I feel comfortable asking any questions to my healthcare provider. |  |  |  |  |  |
| 1. I know where to go when a prescription is ordered. |  |  |  |  |  |
| 1. I am able to let healthcare providers know my preferred pharmacy either by telling them or showing my ID. |  |  |  |  |  |
| 1. I know what to expect after my visit and when I should return if necessary. |  |  |  |  |  |
| 1. If needed, I know how to get specialist care. |  |  |  |  |  |

**Interpretation**

|  | Strongly disagree | Disagree | Neither agree  or disagree | Agree | Strongly  agree |
| --- | --- | --- | --- | --- | --- |
| 1. I am able to request an interpreter at a clinic, pharmacy, or over the phone. |  |  |  |  |  |

**Medicine**

|  | Strongly disagree | Disagree | Neither agree  or disagree | Agree | Strongly  agree |
| --- | --- | --- | --- | --- | --- |
| 1. I am able to get refills when I finish medicine if necessary. |  |  |  |  |  |
| 1. I am able to pick up prescribed or refilled medicines at a pharmacy. |  |  |  |  |  |
| 1. I am aware of medications that do not require a prescription from a healthcare provider (over-the-counter medicine). |  |  |  |  |  |
| 1. I am able to get medications from a pharmacy that do not require a prescription (over-the-counter medicine). |  |  |  |  |  |
| 1. When I cannot get my medicine on time (for example, prescription is not at a pharmacy, or something is wrong with the prescription or medication), I know what to do or I have someone who can help me. |  |  |  |  |  |

**Medical bills**

|  | Strongly disagree | Disagree | Neither agree  or disagree | Agree | Strongly  agree |
| --- | --- | --- | --- | --- | --- |
| 1. I am able to understand medical bills (either myself or using a translating app) or I have someone who can help me understand medical bills. |  |  |  |  |  |
| 1. I know how to pay medical bills when I need to. |  |  |  |  |  |
| 1. If there are any medical billing errors or insurance declines to pay my bills, I am able to address the issues or I have someone who can help me address the issues. |  |  |  |  |  |

**Preventive care**

|  | Strongly disagree | Disagree | Neither agree  or disagree | Agree | Strongly  agree |
| --- | --- | --- | --- | --- | --- |
| 1. People at certain ages need to get certain tests to check their bodies for possible illnesses like cancer, even if they don’t feel sick. |  |  |  |  |  |
| 1. Vaccinations are effective in preventing some diseases. |  |  |  |  |  |
