# Supplementary material for: Developing and validating a HEalthCare NAvigation Competency (HECNAC) Scale for refugees in the United States
Source: PLoS One. 2025 Jan 30;20(1):e0314057. doi: 10.1371/journal.pone.0314057 (PMC11781618; doi:10.1371/journal.pone.0314057)
Supplement: Attachment — Submitted filename: Reviwers comments.docx [file pone.0314057.s004.docx]

Dr. Magdalena Szaflarski

Editor

PLOS ONE

Dear Dr Szaflarski,

Thank you for the opportunity to invite me reviewing this paper titled” Developing and validating a HEalthCare NAvigation Competency (HECNAC) Scale for refugees in the United States”. It is a very interesting topic and the HECNAC scale might have an important role in supporting with healthcare navigation and tailored interventions as well as improve accessibility in refugees’ populations in US, and perhaps can be adapted in many other Western countries for other migrants’ population. Here is a list of detailed comments:

Abstract: Too many background information, and more methods and results are needed such as the description of panels’ characteristics (e.g., number of panels, professions etc.). Also, domains of the scale can be examined in full.

Introduction: It will be interesting to read more about refugees in US, the history, ethnicity, and common languages they spoke at home. Some statistics about this population proportion and background can help to get more understand of the need in developing this scale.

Methods: Is there any other healthcare navigation scale available? It is not clear in Line 74-77 about what frameworks, models and scales available. Please clarify.

Figure 1: please list the stakeholders in the box, e.g., how many refugees, healthcare providers etc. How did those stakeholders been selected? (12 females and 2 males, 6 white) Please clarify.

Results: How long does participant take to complete the scale? Despite the language, are there any feedback regarding the cultural difference and barriers? Please expand more. Will the cognitive interview guide/questions be available? How long are the interviews?

I really like Table 5 the practical considerations. Are they direct quotation from the interviewees? Or has been paraphrased by researcher? If they are quotes, it might be worth to add which ones from the consumers (refuges) and which ones from the healthcare providers. If they have been summarised, please make them more precise, such as the second point is very lengthy.

Discussion and conclusion: this paper is focused on one refugee group who are Dari-speaking Afghans. However, the language Dari is first appeared in mid-Methods, and the Afghan refugee group is not stated elsewhere in the Intro, aim or method sections. Please introduce this population earlier in the paper for clarification.

Thank you again.

Sincerely,

The reviewer
