# Supplementary material for: Developing and validating a HEalthCare NAvigation Competency (HECNAC) Scale for refugees in the United States
Source: PLoS One. 2025 Jan 30;20(1):e0314057. doi: 10.1371/journal.pone.0314057 (PMC11781618; doi:10.1371/journal.pone.0314057)
Supplement: Attachment — Submitted filename: Response letter.docx [file pone.0314057.s006.docx]

Dear Editor and Reviewers for PLOS ONE,

Thank you so much for providing the opportunity to submit this revised manuscript titled ***“Developing and validating a HEalthCare NAvigation Competency (HECNAC) Scale for refugees in the United States”*** to PLOS ONE.

We appreciate your insightful and valuable feedback on our manuscript. It substantially helped enrich the manuscript overall. We have incorporated your suggestions accordingly and highlighted changes within the manuscript using the track-change function. Our responses to each point are below in blue. If you have any additional suggestions or concerns, please kindly let us know.

**Editor and Reviewer Comments and Our Responses:**

**Journal Requirements:**

None proposed.

**Reviewer #1:** All comments have been addressed.

**Reviewer #2:** I appreciate the edits the authors have made to the manuscript. It reads much more clearly now and will be a valuable contribution. I appreciate the context added regarding the influx of Afghan refugees since 2019 as well as the explanation of the outreach in the lead investigator's circle. That said, I still think the context of focusing on Dari speaking Afghans needs much earlier introduction - e.g. in the intro - as a contextual factor, not to minimize the value of the scale developed here nor the importance of this group - likely an understudied group!, but rather because the scale definitively DOES need testing in other refugee groups to ensure the assumptions hold up. On that point, the limitations need to be strengthened, too.

**Response:** Thank you for your suggestion. We moved the section which details the rationale for choosing the Afghan population for testing this scale in the introduction section (lines 63-69). We also strengthened our limitation section based on your suggestion (lines 285-289).
